# A discrete algebraic framework for stochastic systems which yield unique and exact solutions

**DOI:** 10.1016/j.heliyon.2018.e00691

**Published:** 2018-07-17

**Authors:** Michelle Rudolph-Lilith

**Affiliations:** Unité de Neurosciences, Information et Complexité (UNIC), CNRS, Gif-sur-Yvette, France

**Keywords:** Applied mathematics, Statistical physics

## Abstract

Many physical systems exhibit random or stochastic components which shape or even drive their dynamic behavior. The stochastic models and equations describing such systems are typically assessed numerically, with a few exceptions allowing for a mathematically more rigorous treatment in the framework of stochastic calculus. However, even if exact solutions can be obtained in special cases, some results remain ambiguous due to the analytical foundation on which this calculus rests. In this work, we set out to identify the conceptual problem which renders stochastic calculus ambiguous, and exemplify a discrete algebraic framework which, for all practical intents and purposes, not just yields unique and exact solutions, but might also be capable of providing solutions to a much wider class of stochastic models.

## 1 Introduction

The first descriptive mentioning of an intriguing new type of motion governing the behavior of tiny particles, now generally known as “Brownian motion”, can be traced back to the Roman poet and philosopher Titus Lucretius Carus (Lucretius), who already around 60 BCE noticed and described with remarkable accuracy the seemingly random movement of dust in air [1]. Although Lucretius attributed the cause behind this jiggling dance of dust to tiny air currents created by sunlight, he also suggested that collisions between atoms are responsible for its spontaneous nature. It took almost two millennia before observations of this phenomenon, specifically the motion of small organic particles submerged in fluids, appeared again in the literature, e.g. in the works of biologists Stiles and Gleichen (movement of pollen and particles of the ovulum of Zea Mays), Needham, Leclerc (Buffon) and Spallanzani (movement of pollen, active molecules and other organic particles), as well as the botanist Brogniart (movement of pollen-grain; for a more detailed account of the historical experimental background, see [2], [3]). However, the physical explanation of the observed phenomenon remained obscure. Slow progress was finally made in more detailed investigations by Ingen-Housz, who showed that coal dust on the surface of alcohol is subject to the same type of random motion [4], and, independently, the studies of Bywater [5]. Both demonstrated that the observed phenomenon is not restricted to organic materials, but governs the motion of tiny inorganic particles as well, thus opening the door for a more focused approach to delineate its physical causes. It was finally botanist Robert Brown who, standing on a large body of available experimental work, first investigated this seemingly random particle motion in a systematic fashion [2], effectively constructing the foundation on which later the theoretical framework and mathematical description of this phenomenon could be built. For this arguably justifiable reason, in a historical context, the discovery of what later became known as “Brownian motion” is attribution to him.

However, also Brown was unsuccessful in identifying the physical cause of this motion, let alone conceiving of a mathematical description of this phenomenon within the mindset of Newtonian determinism dominating physics at this time. It still took almost a century before Einstein [6], and independently Smoluchowski [7], provided a mechanistic explanation of this random motion, and proposed a mathematical model which allowed for its more rigorous treatment. Today, Brownian motion is cited as a classical and, perhaps, the most simple example of stochastic processes, and its mathematical analysis serves as the primary illustration of what became known as stochastic calculus [8], [9]. However, despite the overwhelming success of stochastic calculus in describing natural phenomena and its many applications far beyond the realms of physics, a conceptually satisfying solution to the core conundrum surrounding it, namely the fact that results of stochastic calculations typically depend on the chosen mathematical convention (e.g., see [9], [10], [11], [12], [13], [14]) is still at large (but see, e.g., [15] Chapter X and [16]).

In this contribution, we set out to explore this dilemma from a perhaps naive, more foundational angle, and assert that its source is rooted in the idealizing assumption of the continuity of physical observables and, resting on this assumption of continuity, the analytic mathematical framework commonly used to describe physical phenomena. Although discretization of physical observables is an accepted and widely employed technique to render mathematical models of natural phenomena finite and, thus, treatable, we will conjecture that only a rigorously discrete and finite mathematical framework can provide a meaningful description of physical measurements as well as basis for the construction of models describing physical reality, at least, but likely not only, in cases where stochastic observables are involved.

Unfortunately, however, to date such a well-developed discrete-algebraic framework remains largely unexplored beyond the focus on mere applicational issues (for examples of such discrete approaches, see [17], [18]). In this contribution, we will therefore exemplify the proposed approach by considering a simple yet not explicitly and unambiguously solvable stochastic model, and demonstrate that, for all practical intents and purposes, an exact solution can be obtained for the system's state variable. Moreover, the presented constructive solution is unique and, thus, devoid of the aforementioned conceptual dilemma which riddles stochastic calculus. With this, we hope to shed some light on the nature of stochastic phenomena, as well as the mathematical language which would be necessary to adequately describe such phenomena.

## 2 Background

Einstein's physical explanation and mathematical description of Brownian motion are often cited as the first stochastic model of a natural phenomenon. Looking at the change of the number of particles per unit volume in finite time intervals [6], however, Einstein's derivation is also widely regarded as only approximative in nature. This arguably unjust verdict does remain untouched even when considering the finite time intervals as being infinitesimally small compared to the time the system is observed, thus translating the original finite and discrete model into one which describes the spatio-temporal distribution of particles in terms of a continuous diffusion equation. Regardless of the approximative nature of Einstein's model of Brownian motion, the associated diffusion equation formed historically the impetus for the development of stochastic calculus. Here, Langevin [19] formulated the first method for generalizing dynamical equations to probabilistic equations by considering differential equations of continuous functions with random terms such as white noise. However, it quickly became clear that the mathematically rigorous treatment of such equations requires the generalization of commonly used classic-analytical concepts, specifically that of the differential and integral, and it took yet another four decades before Itô introduced a rigorous notion of stochastic differentials and integrals [20], [21].

Despite the rigor of Itô's stochastic calculus, it is far from being free of conceptual problems. To illustrate this point, let us consider the random term in Langevin's equation as being a highly irregular and rapidly fluctuating function. In order to give mathematical meaning to an associated stochastic differential equation, this random term must be made subject to stringent restrictions. Among other properties, one has to demand that values taken by the random term at different times are not correlated. Although being a reasonable constraint to impose, mathematically it leads to a *δ*-shaped autocorrelation function and, thus, to an infinite variance of the random term. Physically, there is little justification to support such an assumption when describing natural processes. Unfortunately however, many stochastic models of natural phenomena utilize Gaussian white noise due to its mathematical simplicity, a stochastic process which satisfies on a mathematical level the above requirement, yet remains physically an ideal which cannot have a realization in nature. Indeed, in a recent experimental study [22], it was demonstrated that, due to long-range hydrodynamic correlations, the thermal forces governing the random motion of Brownian particles cannot be characterized by a white noise spectrum, as commonly assumed in stochastic models of Brownian motion. In other experiments (e.g., see [23]), it could be argued that the assumption of white noise is justified, but that such a justification can only be maintained under very specific circumstances ultimately leading to mathematical models of the investigated physical system which no longer respect the system's physical boundaries, an argument which will be further exemplified below.

Viewed from a more mathematical perspective, the seemingly insurmountable conceptual problems one faces when dealing with stochastic systems become even more transparent. Let us consider, as an example, a differential equation with an additive stochastic term f(t) subject to *δ*-autocorrelation, i.e. $\langle f(t)f(t^{\prime })\rangle =\delta (t-t^{\prime })$. Assuming that the associated differential equation describes a physical phenomenon, we must demand it to be integrable, hence the integral

$$\int_{0}^{t}f(s)\;ds=F(t)$$

must both exist and yield a continuous function F(t), called a Wiener process. However, it can be shown that F(t) is not differentiable, thus rendering the original differential equation altogether meaningless in a strict mathematical sense [9].

Itô's contribution to the solution of this conundrum was, instead of dealing with the integrated stochastic term itself, to remain with the formal integral equation and define a stochastic differential

dF(t)=f(t)dt

denoting the increment of the Wiener process F(t). Arguably nothing more than a valid mathematical trick, with this re-interpretation, the above integral takes the form

$$\int_{0}^{t}f(s)\;ds=\int_{0}^{t}dF(s),$$

which is the simplest example of a stochastic Riemann–Stieltjes integral [24] endowed with a mathematically well-defined meaning.

In general, integrals of the form

(1) $$\int_{t_{0}}^{t}g(s,F(s))\;dF(s),$$

where g(t,F(t)) denotes an arbitrary real-valued function which is $C^{1}$ in both *t* and the Wiener process F(t), as well as independent of the behavior of F(t) for future values of *t* (i.e. nonanticipating; see [9], Chapter 4), are treated similar to the classical Riemann integral by partitioning of the integration interval and considering the asymptotic limit of partial (Riemannian) sums. Specifically, let $\left\{t_{i}\},i\in [0,n\right]$ with $t_{i-1}<t_{i}$ and $t_{n}=t$ be a discretization of the interval $\left[t_{0},t\right]$ into *n* equidistant points, and choose for each i>0 a point

(2) $$\tau_{i}=\alpha t_{i}+(1-\alpha )t_{i-1}$$

with 0≤α≤1 residing inside the interval $\left[t_{i-1},t_{i}\right]$. Expressing (1) in terms of an *α*-integral, an approach which generalizes Stratonovich's evaluation of stochastic integrals, it can be shown [25] that the general solution is given by

(3) $$\begin{array}{ccc} \int_{t_{0}}^{t}g(s,F(s))\;dF(s) & = & G(t,F(t))-G(t_{0},F(t_{0}))-\int_{t_{0}}^{t}\frac{\partial G(s,F(s))}{\partial s}\;ds\\ & & +\left(\alpha -\frac{1}{2}\right)\int_{t_{0}}^{t}\frac{\partial^{2}G(s,F(s))}{\partial F{\left(s\right)}^{2}}\;ds, \end{array}$$

where G(t,F(t)) denotes a function such that

$$\frac{\partial G(t,F(t))}{\partial F(t)}=g(t,F(t)).$$

If we set g(t,F(t))=F(t), then (3) yields

(4) $$\begin{array}{c} \int_{t_{0}}^{t}F(s)\;dF(s)\\ \;=\frac{1}{2}\left(F{\left(t\right)}^{2}-F{\left(t_{0}\right)}^{2}\right)+\left(\alpha -\frac{1}{2}\right)(t-t_{0})\\ \;=\left\{ \begin{array}{ccc} \frac{1}{2}\left(F{\left(t\right)}^{2}-F{\left(t_{0}\right)}^{2}\right)-\frac{1}{2}(t-t_{0})\; & \text{for }\alpha =0\; & \text{ (Itô)}\\ \frac{1}{2}\left(F{\left(t\right)}^{2}-F{\left(t_{0}\right)}^{2}\right)\; & \text{for }\alpha =1/2\; & \text{ (Stratonovich)} \end{array}\right. \end{array}$$

which is one of the most-cited examples of stochastic integration in the literature.

The integrals on the right-hand side of (3) are classical Riemann integrals, thus exist and are well-defined for appropriate functions g(t,F(t)). However, the last term renders the stochastic integral somewhat pathologic in a mathematical sense, as the general solution remains dependent on the arbitrary choice of the point (2) at which the argument is evaluated when considering the partial sums, even after taking the asymptotic limit. This is in stark violation of the general tenet that the mathematical description of physical phenomena cannot and must not depend on the chosen mathematical convention, and highlights one of the main problems in the theory of stochastic integrals and, thus, stochastic calculus in general.

Historically, two conventional choices for *α* emerged and are now most-widely used. For α=0, one obtains Itô's original calculus [20], [21], which was found to be both mathematically and technically most satisfying, however, on the expense of differential rules which deviate from those utilized in classical calculus. While the Itô calculus dominates applications in financial mathematics (e.g., see [26], [27], [28]; but see [29]), the last term on the right-hand side in (3), or $\frac{1}{2}(t-t_{0})$ in the example presented in Eq. (4), often eludes a physical meaning. On the other hand, $\alpha =\frac{1}{2}$ yields the well-known Stratonovich calculus [30], an analytic framework which retains the classic rules of calculus and, although being mathematically more difficult to deal with, constitutes the natural choice for the description of physically more realistic phenomena involving stochastic processes with finite correlation time.

To make matters worse, a rigorous link between both established and employed stochastic calculi or, in general, the calculi emerging from an arbitrary choice of α∈[0,1] is still at large, and despite many promising attempts (e.g., see [31], [32], [33]), so far no general rule could be established which dictates which calculus to use for modelling specific stochastic phenomena of physical reality. Perhaps the term “Itô–Stratonovich dilemma” [9], [15], [34], [35] is well chosen, as it suggests a more general problem with our understanding of the nature of mathematical models of stochastic phenomena, and not just the insufficiency of a single model and the particular calculus utilized to adequately describe it. In the remainder of this paper, we will carefully argue that, indeed, the core mathematical language of differential calculus employed in stochastic calculus itself is ill-suited, as it is an idealization which does not, and cannot, reflect physical reality (Section 3). Furthermore, in Section 5 we will exemplify, perhaps naively, a finite algebraic approach which is, by definition, devoid of ambiguities stemming from the choice between different mathematical conventions.

## 3 Analysis

Let us return to Brownian motion as the classical example of a stochastic phenomenon. Einstein's intuition into the physical nature of this phenomenon provided the mechanical explanation of the observed seemingly random movement as being the result of collisions between suspended particles and the atoms or molecules of a fluid whose behavior is governed by the molecular-kinetic theory of heat and thermal equilibrium [6]. In deducing a mathematical formulation of this mechanical model, Einstein argued that certain constraints have to be satisfied. Firstly, the movement of each suspended particle needs to be considered as being independent from the movement of all other particles. Secondly, the movement of each given particle at different times must be assumed as being independent. In order to satisfy the first constraint and, thus, ensure that statistical equilibrium is achieved, the time intervals considered in the mathematical model must be small enough (preferably infinitesimal) compared to the time frame the whole system is observed. However, the second constraint demands the time intervals considered in the mathematical model to be large enough (certainly not infinitesimal) in order to ensure the aforementioned independence of the random movement of each suspended particles at different times. In other words, a given particle must experience collisions which change its path between successive time intervals. Similar constraining arguments can be made for the space variable entering the mathematical model of Brownian motion, as from a physical point of view both the suspended particles and the molecules or atoms of the fluid are spatially extended objects.

Mathematically, a lower bound for spatial and temporal variables naturally yields a description of the phenomenon in terms of finite and discrete, i.e. algebraic, equations, specifically difference equations, as reflected in the original approach of Einstein. However, in order to establish the link to the well-known phenomenon of diffusion, Einstein discarded later in his original work these lower bounds and arrived at a differential formulation of Brownian motion which, as mentioned earlier, set the stage for the development of stochastic calculus by Itô many decades later. In Einstein's defense, we have to stress that the approach of using infinitesimal limits of certain variables in algebraic equations in order to arrive at a mathematical description in terms of differential, hence analytic, equations is rather commonplace. On the other hand, unfortunately, it also needs to be noted that questions like whether such limits, despite being mathematically sound, make sense physically, or to which extent the taking of limits of variables retains the physical meaning of the original model, are often only barely addressed or, worse, simply ignored.

To exemplify the importance of such questions, let us once more take a look at the example of Brownian motion. What does it mean to consider infinitesimal time intervals? As mentioned above, the lower bound of time intervals in the model of Brownian motion is dictated by the requirement that the movement of a suspended particle must be independent when considering consecutive time intervals. As the observed random movement is, on physical grounds, the consequence of collisions between the particle and the fluid's fast-moving constituents, such an independence is certainly only ensured if, within two consecutive time intervals, at least one collision occurs. To retain independence of movement when asymptotically approaching infinitesimally small time intervals, an increasing number of collisions is required, which physically necessitates the fluid in which the Brownian particle is suspended to either have a temperature or a density approaching infinity. On the other hand, if we retain physically plausible properties of the system, i.e. finite density and temperature, then choosing increasingly smaller time intervals must result in a mathematical model whose behavior does no longer capture the random nature of the movement of the Brownian particle. Indeed, for sufficiently small yet still finite time intervals, no collisions between the suspended particle and the fluid's constituents will occur, hence the motion of both the Brownian particles and that of the fluid will be deterministic in the classical Newtonian sense and, thus, exhibit no stochastic behavior. In other words, one could argue that the random nature of the Brownian motion is merely an emergent phenomenon tied to the time scale at which the system is observed, and that a valid mathematical model must respect this scale. Brownian motion is certainly not an isolated case exhibiting this breakdown of the link between physical reality and its mathematical description, but arguments similar to the ones made above can be brought forth for many, possibly all stochastic models of physical phenomena. Either the mathematical limits required to obtain differential formulations of the given stochastic phenomena do not make sense physically, or yield models which no longer reflect the physical reality they were originally constructed to describe.

In defiance of this principal problem, various paths were explored to retain the power of differential calculus for describing stochastic systems. As Itô demonstrated, the classical definition of integrals and differentials can be extended to encapsulate stochastic variables by utilizing limits of finite constructs, such as the Riemannian sum in the case of integrals. However, as we saw in Section 2, the results of this approach typically depend on the way these limits are approached, see Eq. (3), thus violating one of the core pillars of modern physics, namely the requirement of independence of a model describing physical reality from mathematical convention. Fixating on one specific convention, such as either Itô or Stratonovich, does not solve the problem either, as for each possible convention, examples of physical systems can be found whose characteristics are at odds with that of the corresponding mathematical model. Last but not least, one could abolish the requirement of a *δ*-shaped autocorrelation crucial to Itô's original stochastic calculus all together. This certainly moves stochastic differential calculus closer to its classical counterpart (e.g., see [9], [15], [36]), but opens up a whole new Pandora's Box of demonstrating the mathematical rigor of the resulting calculus. Finally, and in conjunction with this idea, it certainly is conceivable to retain the differential framework at the base of stochastic calculus, along with its well-documented advantages, and somehow “cure”, in a mathematically rigorous way, the resulting formalism from its conceptual shortcomings. Specifically, fully abandoning the notion of white noise in favor of colored noise with finite correlation times, or introducing new stochastic processes which better account for the microscopic reality of a given physical phenomenon, such as telegrapher's noise [37] and its generalization (which was shown to account for the finite velocities of Brownian particles, see [38]), is a road already well-explored. Unfortunately, however, along this road one often looses the advantages of simplicity and a more general applicability of the generated models, as the latter need to account for, and require, a deep knowledge of the microscopic reality of the given physical situation.

Another and certainly more radical option is to give up on the idea of defining a viable differential calculus suitable for describing stochastic systems altogether. The justification of such an approach is inherently linked to the epistemological problem of conception and validation of models of physical reality, and, in the wider sense, to the theory of measurement of physical observables. Let us define as “physical observables” all variables and parameters entering models of physical phenomena which can be experimentally probed and, thus, are accessible through the process of measurement. Then we can assert three intuitive principles for the construction of mathematical models of physical phenomena:

*(1) Physical observables are finite.* Although intuitively viable and upheld by centuries of experimental observations, mathematical models of physical phenomena often violate this law from experience by considering asymptotic or infinitesimal limits which not only see little reflection in reality, but might even break the boundaries of the validity and applicability of a given model.

*(2) Physical observables can only be ascertained with finite precision.* Experimentally probing physical reality is always subject to noise and uncertainties which limit the precision with which physical observables can be known. But rather than being a nuisance, this principal limit of how precise we can measure and, more generally, how much we can know about nature at any given time might prove an advantageous ingredient in the conception of a mathematical framework better suited to describe reality.

*(3) Physical observables exhibit lower and upper bounds marking the validity of a given model.* It is an acknowledged fact that each given model of a physical phenomenon is and can only be valid within certain bounds of its observables. Considering asymptotic or infinitesimal limits of physical variables in order to arrive at a formulation in terms of differential calculus, however, might breach these bounds and unjustly extend the scope of a given model beyond its applicability, as exemplified above in the case of Brownian motion.

The mathematical framework used for describing physical phenomena must, or at least should, respect these core principles as they encapsulate what can be known, and thwart stepping on the treacherous ground of idealizations whose validation and experimental verification is hard or even impossible to achieve. Moreover, mathematical models of physical reality must, or at least should, never leave the scope of their applicability, as only this way it is possible to draw viable, unambiguous and predictive conclusions one can work with and build upon. We argue that a discrete and finite mathematical framework under the umbrella of algebraic constructivism encapsulates, by definition, all of the above principles and, thus, is better suited as mathematical framework for describing physical phenomena, in particular those with emergent stochastic characteristics.

In the remainder of this study, we will exemplify the power of a purely constructive algebraic mindset and approach, using a simple yet non-trivial stochastic system which cannot be solved unambiguously and explicitly in the framework of stochastic calculus. Unfortunately, however, as a rigorous mathematical framework is still in development, we must abstain from generalizations and a presentation of potential applications, but only demonstrate that, in this specific instance, the explicit and exact temporal stochastic evolution of the system's state variable can be obtained.

## 4 Example

In a discrete algebraic framework, ideally, one would start with a strictly algebraic model of a given physical system in terms of recursive equations which, akin to differential equations, describe the incremental evolution of the system in question. The task then would be to solve these recursions explicitly, hence arrive at an “integrated” model which can be used for formulating predictions and establishing the link to experimental observations. However, as the goal of this study is the presentation of an alternative approach to stochastic calculus, we will utilize a model formulated in terms of differential calculus, and explore its treatment within a constructive algebraic framework (Section 5).

### 4.1 The toy model

For the purpose of illustration, let us consider the following first-order stochastic differential equation in x(t):

(5) $$\overset{˙}{x}(t)=a_{1}x(t)+(a_{2}x(t)+a_{3})f(t),$$

where all $a_{i}\in Q:a_{i}\neq 0$ are constants, and f(t) denotes an arbitrary function or stochastic process driving the dynamics of the system. Equation (5) has far-reaching applications. For instance, in the case of f(t) being colored (e.g. Ornstein–Uhlenbeck) noise, the above differential equation is subject to applications in theoretical neuroscience as effective stochastic model of neurons driven by a single multiplicative, i.e. conductance, synaptic noise source (e.g., see [39]). If f(t) is a Gaussian white noise source, then, due to the multiplicative coupling between the state variable x(t) and the noise term, Eq. (5) belongs to the class of primary examples which is typically utilized to illustrate the Itô–Stratonovich dilemma (e.g., see [9], [10], [34], [35]).

As argued earlier, due to the very nature of stochastic processes, the stochastic differential equation (5) does, in general, not allow for an explicit and, more importantly, unique solution within the framework of stochastic calculus. However, if we consider for a moment f(t) as being a smooth function of *t*, then an explicit, formal solution of this differential equation is given by

(6) $$x(t)=x(0){\text{e}}^{I(t)}+a_{3}\int_{0}^{t}f(s){\text{e}}^{I(t)-I(s)}\;ds,$$

where

(7) $$I(t)=\int_{0}^{t}\left(a_{1}+a_{2}f(s)\right)\;ds.$$

**Figure 1:**
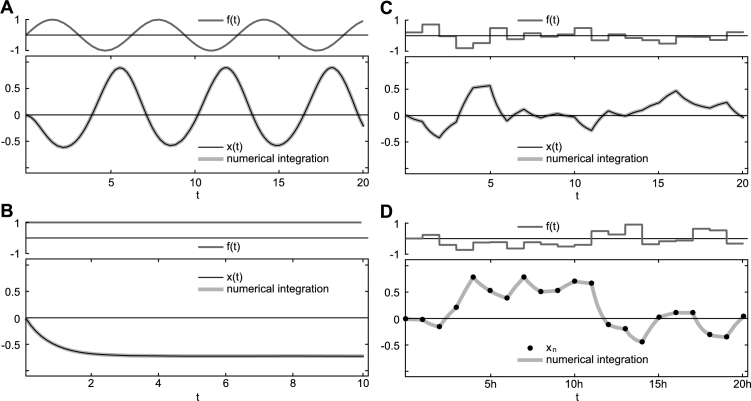
Representative examples of solutions of the differential equation (5) for various inputs *f*(*t*). Compared are the numerical integration of the original system (grey), the explicit analytical solution ((A)–(C): black) and recursive algebraic solution ((D): dots). (A): Eq. (6) for f(t)=sin⁡(t); (B): Eq. (8) for *f*(*t*)=*const*; (C): Eq. (10) for *f*(*t*)=*f*_*t*_ = *const* for *t* ∈ (*t*,*t* + Δ*t*]; (D): Eq. (11) for *f*_*n*_ = *const*∀n∈N. Model parameters: *a*_1_ = −1, *a*_2_ = −0.4 for (A)–(C) and *a*_2_ = −1 for (D), *a*_3_ = −1, *x*(0)≡*x*_0_ = 0; (A): f(t)=sin⁡(t); (B): *f*(*t*)=1; (C) and (D): *f*_*t*_ and *f*_*n*_ were chosen from a normal distribution with mean 0 and standard deviation 0.4. Numerical evaluations were performed using Mathematica 10 [40], with a precision goal of 10^−100^, h=Δt=1 in all cases. Numerical integration of the original set of differential equations (5) was performed using NIntegrate with default settings, ensuring the precision goal and, thus, an integration step ≪h.

As an example, Figure 1A illustrates for f(t)=sin⁡(t) the numerical evaluation of this solution, and compares the latter to the numerical integration of the original differential equation (5).

### 4.2 The explicit analytic solution

Although (6) provides, under the weak assumption of smooth and integrable f(t) in the interval [0,t], an explicit solution of (5), a closed analytic form for arbitrary f(t) can, in general, not be obtained. A case which does, however, allow for such a solution and will play a crucial part in the remainder of this study, is given if we assume f(t) as being piecewise constant. Specifically, let $f(t)=f_{t}$ be constant in the left-open finite interval (t,t+Δt], Δt>0. For notational simplicity but without loss of generality, we will first consider the interval (0,Δt]. In this case, Eq. (6) directly yields

(8) $$x(t)=x_{0}{\text{e}}^{(a_{1}+a_{2}f_{0})t}+\frac{a_{3}f_{0}}{a_{1}+a_{2}f_{0}}\left({\text{e}}^{(a_{1}+a_{2}f_{0})t}-1\right)$$

∀t∈(0,Δt]. A representative example is illustrated in Figure 1B.

Before proceeding, we would like to stress that although the assumption made here, namely that all parameters and variables in the considered model are elements of Q, and not R, might appear as irrelevant subtlety, this subtlety is essential both mathematically for remaining within the discrete algebraic, i.e. finite, framework which forms the basis of the approach presented in the next section, as well as conceptually for remaining within a physical framework consistent with the three intuitive principles argued for in Section 3. A consistent restriction to Q not just ensures that the solution for x(t) presented above, which will be used in the remainder of this article, is valid and finite, but also weakens or even absorbs conditions imposed on differential equations to ensure their solvability within the framework of classical differential calculus.

## 5 Results

As shown in the last section, under certain conditions, an explicit closed-form solution of the differential equation (5) can be obtained. Unfortunately however, this does not include f(t) being a stochastic variable. In this case, numerical integration of the original differential equations provides, so far, the only viable and generally trusted approach. Here, a sizeable variety of techniques is readily available, each of which is naturally based on discretization by transforming a original differential equation into a difference equation, and assessing the latter numerically. Such a discretization is justified, and typically yields results approximating what is considered the “exact” solution, if certain mathematically weak requirements are met. Specifically, and perhaps most importantly, the temporal resolution employed in the numerical integration must be higher than the smallest time constant occurring in the system in order to faithfully capture the system's dynamical properties.

The approach presented here, however, will deviate from such a naive numerical discretization. As we saw above, Eq. (8) present an analytically exact solution of (5) under the condition of constant input during the time interval Δ*t*. One can now argue that restricting to arbitrary yet finite Δ*t*, and assuming a constant input f(t) in each consecutive time interval, constitutes the basis for a justifiable approach to solve (5), even in cases in which f(t) is being sampled from a stochastic process. To that end, we will generalize in this section the exact solution presented above to arbitrary *t*, and deduce an algebraic recursion which delivers, for all practical intents and purposes, the analytically exact temporal development of the state variable x(t) in discrete time steps under piecewise constant yet arbitrary inputs f(t). Moreover, we will demonstrate that, to a certain extent, this algebraic recursion can be “integrated” to yield an explicit solution valid and exact for every *t*.

### 5.1 The recursive algebraic solution

As pointed out above, the solution of (5) for constant $f(t)=f_{0}\in Q$ and boundary values x(0)∈Q obtained in the previous section, Eq. (8), is valid ∀t∈(0,Δt] with Δt>0. With an appropriate choice of boundary values, this solution can be easily generalized to arbitrary t>0. To that end, we assume that f(t) is a piecewise constant function with constant step width Δt∈Q:Δt>0, defined as

(9) $$f(t+s)=f_{t}=const$$

with $f_{t}\in Q,s,t\in Q:s\in (0,\Delta t],t=n\Delta t,n\in N$. In this case, the solution given in (8) generalizes to

(10) $$x(t+s)=x(t){\text{e}}^{(a_{1}+a_{2}f_{t})s}+\frac{a_{3}f_{t}}{a_{1}+a_{2}f_{t}}\left({\text{e}}^{(a_{1}+a_{2}f_{t})s}-1\right),$$

where s∈(0,Δt]. As $f_{t}$ can take arbitrary values, Eq. (10) provides a valid and piecewise exact solution of (5) in intervals of length Δ*t* even in cases where f(t) describes a discrete-time stochastic process. Figure 1C visualizes a representative example in which values of $f_{t}$ at different times *t* are drawn from a normal distribution, thus describing a discrete-time stochastic process which, in the statistical limit, resembles a Gaussian white noise process.

Before proceeding towards a recursive algebraic form of the solution presented above, it is important to note that the Δ*t* occurring here plays a role which fundamentally differs from the notion of “step size” utilized in numerical integration schemes. As pointed out earlier, the latter serves as a mere quantitative measure of the numerical discretization at the level of differential equations, and as such is subject to constraints to ensure not only the numerical stability of the solution, but also a satisfactory numerical accuracy. However, irrespective of the size of Δ*t*, numerical integration approaches can, in general, only deliver approximate solutions which typically accumulate numerical errors. This is not the case in the solution presented above. Indeed, Eq. (10) provides the exact analytic form of the solution of the system of differential equations (5) under the assumption (9), with Δ*t* serving as a quantifier for the level of discretization of this solution, and not of the underlying differential equations. Indeed, due to the definition of Δ*t*, this discretization is dictated solely by the “sampling rate” with which the input f(t) drives the system, i.e. the accuracy with which the driving force is or can be known. Thus, in contrast to the notion of step size in numerical integration schemes, Δ*t* is endowed with a direct link to the process of measurement, hence physical reality, and not mathematical conditions of stability and accuracy. Recalling the fact that each physical observable is subject to constraints regarding its precision, we accept that each experimental observation can only deliver a finite and discrete set of values for a probed observable. Such a set can always be expressed in a form similar to (9). In this sense, the solution presented in Eq. (10) and, more generally, the type of discretization explored here are consistent with the principles highlighted in Section 3, with Δ*t* quantifying the limitations imposed by the process of experimental measurement.

This decoupling of numerical precision and the role of Δ*t* constitutes the conceptual basis which does now allow to deduce a recursive algebraic form of (10). Indeed, this equation is already somewhat recursive in the sense that the solution at t+s with s∈(0,Δt] depends solely on the solution at time *t*, and the constant value of $f(t)=f_{t}$ in the interval (t,t+Δt]. The value of the state variable x(s) within open intervals (t,t+Δt) thus becomes irrelevant due to the considered discretization, and can be discarded. With this, *t* acts as a mere label, and, without loss of generality, can be replaced by n∈N,n≥0. Equation (10) then takes the discrete, specifically recursive, algebraic form

(11) $$x_{n+1}=x_{n}{\text{e}}^{(a_{1}+a_{2}f_{n})h}+\frac{a_{3}f_{n}}{a_{1}+a_{2}f_{n}}\left({\text{e}}^{(a_{1}+a_{2}f_{n})h}-1\right),$$

where h:=Δt∈Q for notational convenience. A representative example of the recursive algebraic solution is presented in Figure 1D.

It is important to note that, due to their recursive nature, Eq. (11) describes the exact incremental evolution of the system in finite and strictly positive steps h∈Q. In this sense, a recursive algebraic solution is conceptually equivalent to a formulation in terms of differential equations, which describe the evolution of a system in infinitesimal steps *dt*. Moreover, the algebraic nature of (11) ensures that the solution is not only unique, but also finite for arbitrary h and *n*, irrespective of the convergence properties for n→∞. With this in mind, we will next recover the explicit algebraic form for $x_{n}$, thus move towards an “exact”, within the context of our approach, solution of (5).

### 5.2 The explicit algebraic solution

Although (11) appears to have a somewhat delicate mathematical form, it belongs to the class of linear inhomogeneous recurrence relations for which a whole host of techniques is readily available (e.g., see [41]). In order to apply the latter, we first introduce for notational convenience

(12) $$\begin{array}{ccc} A_{n} & := & \exp\left[(a_{1}+a_{2}f_{n})h\right],\\ B_{n} & := & \frac{a_{3}f_{n}}{a_{1}+a_{2}f_{n}}\left({\text{e}}^{(a_{1}+a_{2}f_{n})h}-1\right). \end{array}$$

With this, Eq. (11) takes the simpler recursive form

(13) $$x_{0}=const,x_{n+1}=A_{n}x_{n}+B_{n},n\geq 0.$$

In what follows, we will solve (13) by utilizing what could be termed “operator approach”, as $A_{n}$ acts as operators on the state variable $x_{n}$ at step *n*, thus evolving the system to step n+1.

Successive application of $A_{i}$ for i∈[0,n−1] in Eq. (13) yields the explicit expression

(14) $$x_{n}=\left(\prod_{i=0}^{n-1}A_{i}\right)x_{0}+\sum_{i=1}^{n-1}\left(\prod_{j=i}^{n-1}A_{j}\right)B_{i-1}+B_{n-1}$$

for the state variable $x_{n}$ with n∈N,n≥1. This expression can be further simplified by noting that

$$\prod_{i=0}^{n-1}A_{i}=\exp\left[\sum_{i=0}^{n-1}(a_{1}+a_{2}f_{i})h\right]=\exp\left[na_{1}h+a_{2}F_{n-1}h\right]$$

and

$$\prod_{j=i}^{n-1}A_{j}=\frac{\prod_{j=0}^{n-1}A_{j}}{\prod_{j=0}^{i-1}A_{j}}=\exp\left[(n-i)a_{1}h+a_{2}(F_{n-1}-F_{i-1})h\right].$$

Here, we introduced the linear (unweighted) sum over all previous inputs

(15) $$F_{n}:=\sum_{i=0}^{n}f_{i},$$

which obeys the linear recursive relation

(16) $$F_{0}=f_{0},F_{n+1}=F_{n}+f_{n+1},n\geq 0.$$

This leaves us finally with the exact explicit algebraic solution for x(t), Eq. (5), at discrete times t=nh,n∈N:n≥1 in form of

(17) $$\begin{array}{ccc} x_{n} & = & {\text{e}}^{(n-i)a_{1}h+a_{2}(F_{n-1}-F_{i-1})h}x_{0}\\ & + & \sum_{i=0}^{n-1}\frac{a_{3}f_{i}}{a_{1}+a_{2}f_{i}}\left({\text{e}}^{(a_{1}+a_{2}f_{i})h}-1\right){\text{e}}^{(n-i-1)a_{1}h+a_{2}(F_{n-1}-F_{i})h}. \end{array}$$

Equation (17) is interesting in various respects. Firstly, and most importantly, we note that, although this expression is explicit in the initial state variable $x_{0}$, it still contains a recursive term $F_{n}$ whose values depend on the full history of the inputs $f_{n}$ up to step *n*. The presence of this term, however, is neither surprising nor conceptually at odds with the result obtained when tackling the original differential equation (5) within the confines of differential calculus, i.e. an analytic approach. Integrating a differential equation, in fact the very concept of an “integral” in standard analysis, is equivalent to an infinitesimally-paced summation over all functional values of the integrand. Although in the case of (5) with stochastic term f(t) such an integration is, in general, not possible without ambiguities (see Section 2), a finitely-paced summation within a discrete framework can be performed, as demonstrated above, leading to the presence of nonlinear yet finite and well-defined terms reflecting the “integrated” history of the system, specifically its driving input.

**Figure 2:**
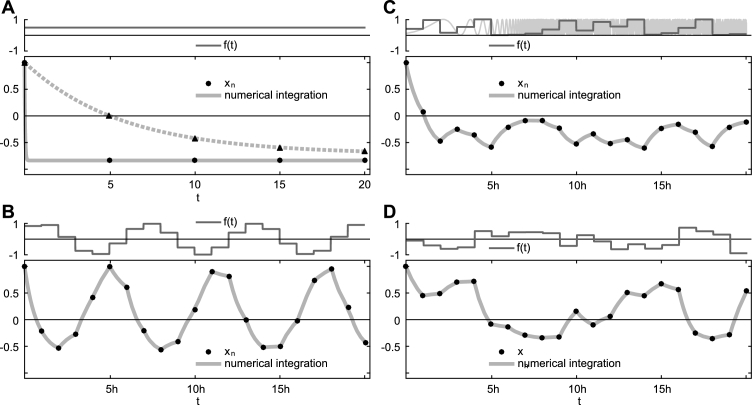
Representative examples of explicit algebraic solutions of the differential equation (5) for various inputs *f*(*t*). Compared are the numerical integration of the original system (solid and dashed), and the explicit algebraic solution (dots and triangles), Eq. (17). (A): Constant input (*f*_*n*_ = *const* ∀*n* ≥ 0) to systems with different intrinsic time constants. Despite differences in the internal dynamics, the algebraic solution allows to calculate accurately the state variable in large intervals (here h=5); (B): $f_{n}=\sin[(n+1)h]$. In this case, $F_{n}$ in Eq. (15) allows for an explicit representation; (C): Discrete-time stochastic process modelled by the logistic map in the chaotic regime, Eq. (21), with *f*_0_ = 0.4; (D): Discrete-time stochastic process with inputs *f*_*n*_ drawn from a normal distribution with mean 0 and standard deviation 0.4. Model parameters: (A): *x*_0_ = 1, *f*_*n*_ = 0.5 ∀*n* ≥ 0, dashed: *a*_1_ = −0.01, *a*_2_ = −0.05, *a*_3_ = −0.05, solid: *a*_1_ = −1, *a*_2_ = −10, *a*_3_ = −10; (B)–(D): *a*_1_ = −1, *a*_2_ = −0.5, *a*_3_ = −1, *x*_0_ = 1. Numerical evaluations were performed using Mathematica 10 [40], with a precision goal of 10^−100^, h=Δt=5 (A) and h=Δt=1 (B–D). Numerical integration of the original set of differential equations (5) was performed using NIntegrate with default settings, ensuring the precision goal and, thus, an integration step ≪h.

Secondly, and as already detailed above, the parameter h has an interpretation which differs from the notion of “step size” in the numerical integration of differential equations. The latter is not just crucial for the stability but also the accuracy of the numerical solution, especially when considering systems whose intrinsic dynamics is fast, in which case the step size in classical numerical integration schemes must be chosen small enough to faithfully capture the system's dynamical properties. In contrast, the parameter h in (17) is only constraint by the sampling rate of the input $f_{n}$, thus linked to experimental limitations or the resolution of the discrete-time stochastic process used in numerical simulations. To illustrate this point, Figure 2A compares the case of constant input, $f_{n}=const\;\forall n\geq 0$, in two systems with fast and slow internal dynamics. Utilizing classical numerical integration methods, such as Euler or Runge–Kutta, requires an integration step smaller than the typical time constant of the given system. Specifically, in the example visualized, the fast system's time constant is of the order of 0.01 (x(t) decays rapidly to its asymptotic value; Figure 2A, solid), whereas the slow system's x(t) (Figure 2A, dashed) enjoys a slow decay of order 10. In order to capture the intrinsic dynamics of the fast system and ensure numerical stability of the solution, the integration time step required must be smaller than 0.01, while in the latter case a time step short of 10 would suffice. In contrast, (17) yields precise solutions (Figure 2A, dots and triangles) even if h, i.e. the step size after which the corresponding equations are numerically evaluated, is chosen much larger than the smallest time constant occurring in the original fast system, suggesting that h is now decoupled from the intrinsic dynamical properties of the system in question. Indeed, h appears to be solely determined by the driving input (in the given example a constant function) and could be chosen much larger without impairing the numerical precision of the result, as (17) only requires evaluation when this input changes. However, a more thorough investigation of this arguably interesting aspect of the presented discrete algebraic framework is required for a justifiable generalization, and lies outside the scope of this study.

### 5.3 A simple application

As noted above, the algebraic solution (17) is explicit in the state variable $x_{n}$, but contains the term $F_{n}$ which is subject to the linear recursion (15). In many cases, this recursive element can be made explicit. For instance, considering periodic inputs

(18) $$f_{n}=\sin[(n+1)h],$$

it can easily be shown that $F_{n}$ takes the explicit form

(19) $$F_{n}=\frac{\sin[\frac{n+1}{2}h]\sin[\frac{n+2}{2}h]}{\sin[\frac{1}{2}h]}.$$

With such an explicit expression, Eq. (17) can be further simplified, and an illustrative example utilizing (18) is shown in Figure 2B.

In the case of discrete-time stochastic inputs, one generally remains with the necessity to recursively evaluate all coefficients and auxiliary variables entering (17). However, also here, in some specific instances, simplifications are possible. A classical example of stochastic inputs is given by pseudo-random numbers modelled according to a nonlinear discrete map serving as random number generator, such as the logistic map

(20) $$f_{0}\in [0,1],f_{n+1}=af_{n}(1-f_{n}),n\geq 1$$

in its chaotic regime (a=4). Here, an explicit solution of (20) is known and given by

(21) $$f_{n}=\frac{1}{2}\left(1-\cos[2^{n}\gamma ]\right),$$

where $\gamma =\arccos[1-2f_{0}]$. Together with a finite power expansion of this solution, which is also available for general values of *a* (see [42]), $F_{n}$ can be expressed explicitly. A representative example utilizing the explicit solution of the logistic map is shown in Figure 2C. However, in the general case of genuine discrete-time stochastic inputs $f_{n}$ (see Figure 2D for an illustrative example), no simplification is possible, and (17), despite providing an explicit and exact finite solution for the state variable $x_{n}$, remains with the recursive term $F_{n}$.

## 6 Conclusions

In this article, we elaborated on some conceptual issues surrounding stochastic calculus, in particular its validity and faithfulness in representing physical reality, and explored the possibility of a finite and discrete algebraic approach for describing stochastic systems. We exemplified the proposed approach using a prototype stochastic model which has applications in a variety of fields. It was demonstrated that, within a discrete algebraic framework, not just an exact solution of the stochastic temporal evolution of the model's state variable can be obtained, but that this solution is, by construction, also free from the ambiguities which typically riddle classical stochastic calculus.

Although the notion of an “exact solution” utilized here must be taken with extreme care, we argue that all experimental and computational approaches naturally impose stringent albeit fluent limitations on the precision with which physical observables can be known or should be treated. The notion of “exact” used in this study must be understood, and is justifiable, within the context of these principal epistemological limitations, and differs from that utilized when qualifying the accuracy of solutions obtained by utilizing numerical integration methods. In this sense, the solution of the differential equation (5) presented here yields analytically exact values for the state variables at discrete time intervals, with a discretization dictated solely by the sampling rate of the driving input. The proposed algebraic approach is general and applicable to a larger class of stochastic systems as long as this discretization remains finite and is not made subject to asymptotic evaluations. We assert and hopefully favourably argued, however, that the condition of finiteness is in accord with our experience of physical reality. In fact, we hope to have demonstrated with the example of Brownian motion, that an approach which uses asymptotic or infinitesimal limits to arrive at a differential or, more general, analytic description of a physical phenomenon might no longer reflect physical reality or the characteristics of the phenomenon in question, thus rendering itself questionable as descriptive and predictive vessel of reality.

Indeed, arguing from the perspective of physical reality, we identified differential calculus and its underlying analytical framework as an idealization which does break the link between model and reality, at least in cases where stochastic observables or systems with stochastic dynamics are involved. The finite discrete algebraic framework proposed here does respect three fundamental principles of measurement theory, namely that each physical observable is finite, can only be ascertained with finite precision, and exhibits lower and upper bounds marking the limits of the applicability of a given model. Thus, we argue that an algebraic framework does serve as a more suitable mathematical basis for describing physical reality. Although a rigorous mathematical foundation of such an algebraic calculus akin to differential calculus is still mostly missing, we hope to have demonstrated the potential power of a discrete and finite mindset in helping to deal with stochastic models.

However, we must also stress that the utilization of a strictly finite algebraic framework comes at a hefty price. Within such a framework, powerful analytic notions such as “integral” or “differential” have neither conceptual nor direct applicable meaning. Thus, before abandoning analytic and differential calculus as descriptive tools, we must address the question whether analysis, or the finite and discrete algebraic mathematical framework promoted here, are more in tune with physical reality, whether physical reality has a continuous or discrete makeup. Although a contribution to this question on a philosophical level lies outside the scope of this study, we note that, throughout the history of science, this question played and continues to play a central role (e.g., see [43] for a review of philosophical elaborations and historical notes). Unfortunately however, with experimental findings covering both sides of the aisle (e.g., see [44], but see [45]), a definite answer is still at large.

Given the conceptual issues addressed in this article, we ask the Reader to open-mindedly consider the possibility of a discrete makeup of reality itself, and indulge in the far-reaching consequences such a possibility will carry for our mathematical description of nature. Although, at this point in history, the assumption of a discrete and finite makeup of our world is still a matter of mere believe not too different from the assumption of the actual existence of infinity or Cantor's real numbers, we argue that, in lack of any viable proof demonstrating the existence of a truly continuous or infinite real-existing physical system, the assumption of a discrete makeup of reality is fully in accord with experimental observations, logically reasonable and justifiable on epistemological grounds. If indeed found to be true, we must eventually, or ultimately, reject the powerful blue-colored analytical toolset of an ideal real number line in favor of the red pill of discrete and finite (or effinite, see [46]) algebraic constructivism. With this study, we hope to have argued that such a framework might indeed prove useful in arriving at a finite, exact and unambiguous description not just of stochastic phenomena.

## Declarations

### Author contribution statement

Michelle Rudolph-Lilith: Conceived and designed the experiments; Performed the experiments; Analyzed and interpreted the data; Contributed reagents, materials, analysis tools or data; Wrote the paper.

### Funding statement

This research did not receive any specific grant from funding agencies in the public, commercial, or not-for-profit sectors.

### Competing interest statement

The authors declare no conflict of interest.

### Additional information

No additional information is available for this paper.
